# Impact of racial, ethnic, and socioeconomic disparities on presentation and survival of HCC: A multicenter study

**DOI:** 10.1097/HC9.0000000000000477

**Published:** 2024-10-10

**Authors:** Nicole E. Rich, Patricia D. Jones, Hong Zhu, Tanushree Prasad, Amy Hughes, Sandi Pruitt, Caitlin C. Murphy, Karim Seif-El-Dahan, Darine Daher, Gloria Figueroa, Stephanie Castaneda, Lisa Quirk, Michael Gonzales, Osiris Carranza, Samantha Bourque, Nargis Baset, Adam C. Yopp, Amit G. Singal

**Affiliations:** 1 Department of Internal Medicine, UT Southwestern Medical Center, Dallas, Texas, USA; 2 Harold C. Simmons Comprehensive Cancer Center, UT Southwestern Medical Center, Dallas, Texas, USA; 3 Department of Internal Medicine, Parkland Health, Dallas, Texas, USA; 4 Department of Internal Medicine, Division of Digestive Health and Liver Diseases, University of Miami Miller School of Medicine, Miami, Florida, USA; 5 Sylvester Comprehensive Cancer Center, University of Miami Miller School of Medicine, Miami, Florida, USA; 6 Department of Public Health Sciences, Division of Biostatistics, University of Virginia School of Medicine, Charlottesville, Virginia, USA; 7 Peter O’Donnell Jr. School of Public Health, UT Southwestern Medical Center, Dallas, Texas, USA; 8 Department of Health Promotion and Behavioral Sciences, University of Texas Health Science Center at Houston School of Public Health, Houston, Texas, USA; 9 Department of Surgery, UT Southwestern Medical Center, Dallas, Texas, USA

**Keywords:** inequity, disadvantage, liver cancer, deprivation, race and ethnicity

## Abstract

**Background:**

Racial and ethnic disparities have been reported for HCC prognosis, although few studies fully account for clinically important factors and social determinants of health, including neighborhood socioeconomic status.

**Methods:**

We conducted a retrospective multicenter cohort study of patients newly diagnosed with HCC from January 2010 through August 2018 at 4 large health systems in the United States. We used multivariable logistic regression and cause-specific Cox proportional hazard models to identify factors associated with early-stage HCC presentation and overall survival.

**Results:**

Of 2263 patients with HCC (37.6% non-Hispanic White, 23.5% non-Hispanic Black, 32.6% Hispanic, and 6.4% Asian/other), 42.0% of patients presented at an early stage (Barcelona Clinic Liver Cancer stage 0/A). In fully adjusted models, there were persistent Black-White disparities in early-stage presentation (OR: 0.63, 95% CI: 0.45–0.89) but not Hispanic-White disparities (OR: 0.93, 95% CI: 0.70–1.24). Median survival was 16.2 (IQR: 5.8–36.8) months for White patients compared to 15.7 (IQR: 4.6–34.4) months for Hispanic, 10.0 (IQR: 2.9–29.0) months for Black, and 9.5 (IQR: 3.4–31.9) months for Asian/other patients. Black-White disparities in survival persisted after adjusting for individual demographics and clinical factors (HR: 1.30, 95% CI: 1.09–1.53) but were no longer observed after adding HCC stage and treatment (HR: 1.05, 95% CI: 0.88–1.24), or in fully adjusted models (HR: 0.97, 95% CI: 0.79–1.18). In fully adjusted models, Hispanic-White (HR: 0.87, 95% CI: 0.73–1.03) and Asian/other-White (HR: 0.85, 95% CI: 0.63–1.15) differences in survival were not statistically significant, although patients in high-SES neighborhoods had lower mortality (HR: 0.69, 95% CI: 0.48–0.99).

**Conclusions:**

In a multicenter cohort of patients with HCC, racial and ethnic differences in HCC prognosis were explained in part by differences in tumor stage at diagnosis and neighborhood SES. These data inform targets to intervene and reduce disparities.

## INTRODUCTION

HCC is the third leading cause of cancer-related death worldwide and a leading cause of mortality in patients with cirrhosis. HCC mortality continues to rise in the United States, and it is projected to surpass colorectal cancer to become the third leading cause of cancer-related death in the United States by the year 2035.^1^ HCC disproportionally affects underserved populations, with higher age-specific incidence rates among racial and ethnic minority populations (including Black, Hispanic, American Indian/Alaskan Native, and Asian/Pacific Islander individuals) compared to non-Hispanic White individuals. Across all racial and ethnic groups,^2^ incidence rates are higher among those of low neighborhood socioeconomic status (nSES) compared to higher nSES counterparts.

Health disparities are complex and driven by multilevel factors, including distal (eg, health systems and policies), intermediate (eg, neighborhood-level social, physical contexts, and health care factors), and proximal (eg, patient demographics, clinical factors, and tumor biology) determinants.^3^ These disparities extend across the cancer care continuum, including screening, timely diagnostic evaluation, and receipt of guideline-concordant treatment.^4^ Failures at any of these steps along the continuum can exacerbate disparities and contribute to downstream differences in overall survival.^5^,^6^ Prior studies have highlighted racial and ethnic disparities in HCC prognosis, with a recent meta-analysis reporting statistically significantly higher mortality among Black patients and lower mortality among Hispanic and Asian patients compared to non-Hispanic White patients.^7^ However, this study and other studies relied upon the use of hospital-based and population-based cancer registry data that lack granular data for known prognostic factors, including liver disease etiology, liver dysfunction, tumor burden, and performance status.^8^,^9^ Other studies have been limited to single-center recruitment, small sample sizes, and a narrow focus on patient-level sociodemographic and clinical factors.^10^,^11^ Finally, no multicenter studies to date have adjusted for factors known to be prognostic in other cancers, such as behavioral/lifestyle, health care utilization, and socioeconomic status.^12^,^13^,^14^ These limitations highlight the need for data from large, multicenter cohorts with racially and ethnically diverse, well-characterized patients.

Better understanding and quantifying racial and ethnic disparities in HCC prognosis is the first step to identifying intervention targets and informing potential strategies to promote health equity. Therefore, our aim was to examine multilevel factors associated with early-stage presentation and overall survival among a large, racially and ethnically diverse cohort of patients with HCC followed in 4 large urban health systems in the United States.

## METHODS

### Study population

We identified consecutive adult patients aged ≥18 years with HCC diagnosed between January 2010 and August 2018 at 4 large health systems in the United States: UT Southwestern Medical Center (UT Southwestern), Parkland Health (Parkland), University of Miami (U Miami), and Jackson Health System (Jackson). UT Southwestern and U Miami are academic tertiary care referral centers; Parkland and Jackson are integrated safety-net health systems for Dallas County, TX, and Miami-Dade County, FL, respectively, caring for large populations of underserved patients with cirrhosis and HCC. Liver transplant services are available at UT Southwestern, Jackson, and U Miami. Parkland does not offer liver transplant services; however, eligible patients can be referred externally for transplant evaluation. A colocated multidisciplinary HCC clinic at UT Southwestern is staffed by the same group of physician providers as the multidisciplinary clinic at Parkland.^15^ At U Miami and Jackson, a core group of physicians deliver most clinical care to patients with HCC using a fluid referral system.

Patients were identified by maintained patient lists (clinic and tumor board lists) as well as by ICD-9 codes, and included if they had treatment-naive HCC at time of presentation to 1 of the 4 health systems. HCC was defined per American Association for the Study of Liver Disease (AASLD) criteria and staged according to the Barcelona Clinic Liver Cancer (BCLC) staging system.^16^,^17^ We excluded: (1) patients without imaging studies and those for whom we could not determine the date of diagnosis and/or baseline tumor burden; (2) patients with a liver mass without characteristic imaging (LI-RADS 5) or histological confirmation; and (3) patients who received treatment at an outside institution before presentation at any of the study sites. The Institutional Review Boards of UT Southwestern and U Miami approved this study.

### Data collection

We manually abstracted patient demographics, clinical history, laboratory data, and imaging results at HCC diagnosis from the electronic health record (EHR) at each study site. Self-reported race and ethnicity were recorded in the EHR and categorized as non-Hispanic White (White), non-Hispanic Black (Black), Hispanic (any race), non-Hispanic Asian or Pacific Islander (Asian), or Other (self-reported “Other” or unknown race and/or ethnicity, and multiracial individuals).^18^ Insurance status at the time of HCC diagnosis was categorized as Medicare, Medicaid, private insurance, other (eg, medical subsidy plan at Parkland which covers HCC surveillance and treatment outside of liver transplantation and a similar plan at Jackson), or no insurance. Clinical history included health behaviors that impact HCC risk or prognosis (eg, alcohol use [current/prior heavy, current social use, or never] and smoking history [current, former, and never], which were obtained through manual review of the EHR), comorbid conditions, liver disease etiology, degree of liver dysfunction, and HCC-directed treatment. Comorbidity was classified using the CirCom score, a cirrhosis-specific comorbidity score that includes chronic obstructive pulmonary disease, acute myocardial infarction, peripheral arterial disease, epilepsy, substance abuse, heart failure, nonmetastatic cancer, metastatic cancer, and chronic kidney disease, as described.^19^ Liver disease etiology was classified as hepatitis C (viremic vs. post-sustained virologic response), hepatitis B, alcohol-associated liver disease, metabolic dysfunction–associated steatotic liver disease, or other.^20^ The degree of liver dysfunction was assessed by the Child-Pugh class, with ascites and HE classified as none, mild or controlled, and severe or uncontrolled. The Albumin-Bilirubin (ALBI) score, which has been demonstrated to add additional prognostic information beyond the Child-Pugh class in patients with HCC, was also calculated.^21^ Health care utilization patterns of interest included the number of primary care visits, the number of hepatology visits, and the receipt of HCC surveillance in the year before HCC diagnosis. HCC surveillance was defined by receipt of abdominal ultrasound, multiphase CT, or contrast-enhanced MRI in the 12-month period preceding HCC diagnosis (excluding the cross-sectional imaging used to confirm HCC diagnosis).

Tumor characteristics included the number of lesions, maximum tumor diameter, vascular invasion, the presence of distant metastases, and BCLC tumor stage at diagnosis. All surgical, locoregional, and systemic HCC treatments were recorded, and the “most definitive” treatment was assigned for each patient according to the following hierarchy: liver transplantation or surgical resection > local ablation > transarterial chemoembolization, transarterial radioembolization or stereotactic body radiation therapy > systemic therapy.

Neighborhood social determinants of health, including census tract-level socioeconomic status and degree of White, Hispanic, and Black segregation, were measured by linking geocoded patient addresses at diagnosis to federal data sets using ArcGIS, a geographic information software system (ESRI). nSES was classified into 5 quintiles (with the lowest being SES-1 and the highest being SES-5) based on the Yost score,^22^ a composite index of census tract-level variables including income, education, and occupation. Residential racial and ethnic segregation was measured using the location of the quotient of racial residential segregation (LQRRS) measure, that is, the ratio of the degree of segregation in a block group relative to the larger metropolitan area.^23^

### Statistical analysis

We compared patient and tumor characteristics by race and ethnicity using ANOVA and chi-square tests for continuous and categorical variables, respectively. Our primary outcomes of interest were early-stage HCC presentation and overall survival. Early-stage HCC was defined as BCLC stage 0/A for our primary analysis.^16^ Because BCLC stage A patients with large unifocal tumors are often not amenable to curative treatment, we also conducted a sensitivity analysis using Milan Criteria to define early-stage HCC. Univariable and multivariable logistic regression defined factors associated with early-stage HCC presentation. Survival analysis was performed by the Kaplan-Meier method, with follow-up time accrued from the date of HCC diagnosis to the date of last follow-up, liver transplantation, death, or end of the study period (September 1, 2018); we used a log-rank test to compare differences in survival by race and ethnicity. Univariable and multivariable cause-specific Cox proportional hazard regression models identified factors associated with overall survival, with transplantation treated as a competing risk. Missing data (for variables with >5% missingness) were addressed using multiple imputation methods.^24^

For both early-stage HCC presentation and overall survival, nested models with an increasing number of covariates were developed. These models were based on a disparities conceptual model,^3^ beginning with patient-level factors, then adding site-level factors, and finally, adding neighborhood-level factors. For early-stage presentation, Model 1 included basic individual demographics (age, sex, race, and ethnicity), health behaviors (smoking and alcohol use), and clinical factors (presence of diabetes, CirCom comorbidity score, liver disease etiology, and the Child-Pugh score). Model 2 adjusted for health care engagement (primary care provider visit in past 1 year and GI/hepatology visit in past 1 year) in addition to model 1 variables. Model 3 included insurance status and hospital site in addition to model 2 variables. Finally, model 4 additionally included neighborhood-level social determinants of health: nSES (Yost quintile) and residential segregation (LQRRS measure). For the overall survival models, we added tumor-related factors after model 1. Thus, model 2 adjusted for the BCLC stage and HCC treatment receipt in addition to model 1 variables. Variables included in the remaining models were the same as those in the early detection models: model 3 added health care engagement (primary care provider visit in past 1 year and GI/hepatology visit in past 1 year), model 4 added insurance status and hospital site, and model 5 added nSES (Yost quintile) and residential segregation (LQRRS measure). Notably, most covariates included in models 1–5 are not available in large administrative data sets and cancer registries. All tests were 2-sided and performed at the 5% significance level. Statistical analysis was performed using SAS 9.4.

## RESULTS

### Patient characteristics

Among 2263 patients with HCC included in the study, 851 (37.6%) were non-Hispanic White, 737 (32.6%) Hispanic, 531 (23.5%) Black, 90 (4.0%) Asian, and 54 (2.4%) other. Due to the small number of patients included in Asian and other subgroups, these were combined as a single group for comparative analyses. Characteristics of patients by race and ethnicity are detailed in Table 1. In brief, the median age of the patients was 61.2 (IQR: 56.3–67.4) years, and the majority were men (76.2%). Insurance type was diverse in the patient sample (31.7% Medicare, 27.7% private, 17.0% Medicaid, and 23.4% either uninsured or covered by a medical subsidy plan). A higher proportion of Hispanic and Black patients had Medicaid coverage (19.1% and 24.9%, respectively) or were uninsured (11.4% and 10.5%, respectively) compared to White patients (11.1% and 5.9%, respectively) (*p* < 0.001). A higher proportion of Hispanic (27.0%) and Black (52.7%) patients lived in neighborhoods in the lowest SES quintile compared to 11.1% of White patients and 12.5% of Asian/Other patients (*p* < 0.001).

**TABLE 1:** Demographic and clinical characteristics, stratified by race and ethnicity

	Non-Hispanic White (n = 851)	Hispanic (n = 737)	Non-Hispanic Black (n = 531)	Asian/other (n = 144)	*p* ^a^
Hospital, n (%)					
Parkland	167 (19.6)	270 (36.6)	313 (59.0)	56 (38.9)	
U Miami	307 (36.1)	231 (31.3)	70 (13.2)	55 (38.2)	<0.001
UTSW	250 (29.4)	71 (9.6)	69 (13.0)	26 (18.1)	
Jackson	127 (14.9)	165 (22.4)	79 (14.9)	7 (4.9)	
Age (y)	61.8 (57.4–68.2)	61 (55.2–68.1)	60.5 (56.1–65.1)	60.9 (55.8–70.9)	<0.001
Sex, female, n (%)	153 (18.0)	207 (28.1)	132 (24.9)	46 (31.9)	<0.001
Insurance, n (%)					
Medicaid	94 (11.1)	141 (19.1)	132 (24.9)	17 (11.8)	
Medicare	309 (36.3)	215 (29.2)	149 (28.0)	44 (30.6)	<0.001
Private	317 (37.2)	174 (23.6)	87 (16.4)	48 (33.3)	
Other^a^	78 (9.2)	122 (16.6)	105 (19.8)	19 (13.2)	
Uninsured	50 (5.9)	84 (11.4)	56 (10.5)	15 (10.4)	
nSES Yost Quintile, n (%)					
1 (Lowest)	94 (11.1)	199 (27.0)	280 (52.7)	18 (12.5)	
2	164 (19.3)	199 (27.0)	110 (20.7)	23 (16.0)	<0.001
3	170 (20.0)	142 (19.3)	57 (10.7)	32 (22.2)	
4	210 (24.7)	107 (14.5)	44 (8.3)	38 (26.4)	
5	172 (20.2)	57 (7.7)	22 (4.1)	30 (20.8)	
LQRRS White segregation, median (IQR)	1.1 (0.7–1.5)	0.5 (0.2–0.9)	0.2 (0.1–0.6)	0.8 (0.5–1.2)	<0.001
LQRRS Black segregation, median (IQR)	0.5 (0.1–1.1)	0.5 (0.1–1.2)	2.6 (1.0–4.3)	0.7 (0.3–1.1)	<0.001
LQRRS Hispanic segregation, median IQR)	0.8 (0.5–1.3)	1.4 (0.9–1.9)	0.9 (0.5–1.5)	1.0 (0.6–1.6)	<0.001
Smoking use, n (%)					
Current	248 (29.1)	108 (14.7)	231 (43.5)	23 (16.0)	<0.001
Former	360 (42.3)	280 (38.0)	166 (31.3)	44 (30.5)	
Never	214 (25.1)	330 (44.8)	129 (24.3)	75 (52.1)	
Alcohol use, n (%)					
Current/prior heavy	417 (49.0)	328 (44.5)	261 (49.2)	37 (25.7)	
Current social use	125 (14.7)	93 (12.6)	100 (18.8)	16 (11.1)	<0.001
Never	309 (36.3)	315 (42.7)	170 (32.0)	91 (62.2)	
Presence of diabetes	278 (32.7)	334 (45.3)	152 (28.6)	46 (31.9)	<0.001
Liver disease etiology, n (%)					
Post-SVR hepatitis C	196 (23.0)	90 (12.2)	67 (12.6)	10 (6.9)	
Viremic hepatitis C	338 (39.7)	227 (30.8)	332 (62.5)	41 (28.5)	
Hepatitis B	35 (4.1)	36 (4.9)	69 (13.0)	54 (37.5)	<0.001
Alcohol-associated	111 (13.0)	159 (21.6)	27 (5.1)	8 (5.6)	
MASLD	90 (10.6)	142 (19.3)	8 (1.5)	11 (7.4)	
Cryptogenic/Other	81 (9.5)	83 (11.3)	28 (5.3)	20 (13.9)	
Underlying cirrhosis	766(90.0)	685 (92.9)	471 (88.7)	115 (79.9)	<0.001
Ascites, n (%)					
None	463 (54.4)	375 (50.9)	338 (63.7)	91 (63.2)	
Minimal/controlled	298 (35.0)	303 (41.1)	152 (28.6)	44 (30.6)	<0.004
Severe/uncontrolled	82 (9.6)	57 (7.7)	39 (7.3)	8 (5.6)	
HE, n (%)					
None	620 (72.9)	517 (70.2)	461 (86.8)	118 (81.9)	<0.001
Minimal/controlled	200 (23.5)	205 (27.8)	61 (11.5)	22 (15.3)	
Severe/uncontrolled	18 (2.1)	12 (1.6)	4 (0.8)	1 (0.7)	
Child-Pugh score, median (IQR)	6.0 (5.0–8.0)	6.5 (5.0–8.3)	5.7 (5.0–7.0)	5.5 (5.0–7.2)	<0.001
Child-Pugh class, n (%)					
Child-Pugh A	425 (50.0)	303 (41.2)	302 (56.8)	84 (58.2)	<0.001
Child-Pugh B	315 (37.0)	312 (42.3)	183 (34.5)	50 (34.9)	
Child-Pugh C	111 (13.0)	122 (16.5)	46 (8.7)	10 (6.9)	
ALBI score	−3.0 (−3.5, −2.3)	−2.7 (−3.3, −2.2)	−3.0 (−3.4, −2.4)	−3.0 (−3.8, −2.4)	<0.001
ALBI grade, n (%)					
1	542 (63.7)	412 (55.9)	385 (67.5)	95 (66.0)	0.001
2	277 (32.6)	289 (39.2)	154 (29.0)	46 (32.0)	
3	32 (3.7)	36 (4.9)	19 (3.5)	3 (2.0)	
MELD score	10 (7–13)	10 (8–14)	9 (7–13)	9 (7–13)	0.007
CirCom, n (%)					
0	483 (56.8)	432 (58.6)	223 (42.0)	77 (53.5)	
1 + 0	162 (19.0)	127 (17.2)	147 (27.7)	31 (21.5)	
1 + 1	70 (8.2)	61 (8.3)	34 (6.4)	12 (8.3)	<0.001
3 + 0	12 (1.4)	5 (0.7)	10 (1.9)	6 (4.2)	
3 + 1	32 (3.8)	47 (6.4)	44 (8.3)	4 (2.8)	
5 + 0	69 (8.1)	47 (6.4)	46 (8.7)	11 (7.6)	
5 + 1	23 (2.7)	18 (2.4)	27 (5.1)	3 (2.1)	
Surveillance within 1 y before HCC diagnosis, n (%)	222 (26.1)	203 (27.5)	99 (18.6)	31 (21.5)	0.002
PCP visit within 1 y before HCC diagnosis, n (%)	100 (11.8)	153 (20.8)	191 (36.0)	26 (18.1)	<0.001
Hepatology visit within 1 y before HCC diagnosis, n (%)	145 (17.0)	114 (15.5)	97 (18.3)	25 (17.4)	0.612
PCP or hepatology visit within 1 y before HCC diagnosis, n (%)	196 (23.0)	192 (26.0)	221 (41.6)	36 (25.0)	<0.001

a Comparisons were made using ANOVA and chi-square tests for continuous and categorical variables, respectively.

Abbreviations: ALBI, albumin-bilirubin score; LQRRS, location of the quotient of racial residential segregation; MASLD, metabolic dysfunction–associated steatotic liver disease; MELD, Model for End Stage Liver Disease; nSES, neighborhood socioeconomic status; PCP, primary care provider; SVR, sustained virologic response; U Miami, University of Miami; UTSW, University of Texas Southwestern.

A higher proportion of Hispanic patients had alcohol-associated liver disease or metabolic dysfunction–associated steatotic liver disease–associated HCC, whereas Black patients comprised the highest proportion of patients with untreated hepatitis C infection (*p* < 0.001). Nearly half (49.3%) of the cohort had Child-Pugh A cirrhosis at the time of HCC diagnosis, with 38.0% and 12.7% having Child-Pugh B and C cirrhosis, respectively. Hispanic patients had a higher median Child-Pugh score compared to White, Black, and Asian/Other patients, a lower proportion of Child-Pugh A cirrhosis (41.2% vs. 50.0%, 56.8%, and 58.2%, respectively; *p* < 0.001), and a higher proportion with ALBI grade 2-3 (44.1% vs. 36.3%, 32.5%, and 34%, respectively; *p* = 0.001). Only one-fourth of the cohort had a history of HE at the time of HCC diagnosis, although nearly half (43.4%) had a history of ascites. Over half of patients had Eastern Cooperative Oncology Group score performance status score 0, 29.5% had Eastern Cooperative Oncology Group score performance status score 1, and 12.0% had Eastern Cooperative Oncology Group score performance status score 2-4. The cohort was otherwise relatively healthy, with 53.7% and 20.6% having CirCom comorbidity scores of 0 and 1+0, respectively. Only 28.5% of patients had a documented primary care provider or hepatology visit in the year before HCC diagnosis, and a minority (24.5%) had HCC surveillance performed in the year before HCC diagnosis.

### Tumor stage at diagnosis and most definitive treatment

Overall, most patients had unifocal HCC at diagnosis, while 16.1% had 2 lesions, 6.6% 3 lesions, and 19.0% had more than 3 lesions and/or infiltrative tumors. Vascular invasion and distant metastatic disease were present in 18.9% and 9.1% of patients, respectively. The median alpha-fetoprotein at diagnosis was 34.7 ng/mL (IQR: 6.7–644.0), with higher levels among Asian and Black patients compared to White and Hispanic patients. Less than half (42.0%) of patients were diagnosed at an early stage, with 20.6% having BCLC stage B disease, 20.7% BCLC stage C, and 16.4% BCLC stage D. Table 2 describes differences in tumor characteristics at diagnosis by race and ethnicity. Overall, 36.9% of Black, 41.0% of Hispanic, 43.8% of Asian/Other, and 45.7% of White individuals presented with BCLC stage 0/A HCC (*p* < 0.001). Most definitive HCC treatment differed by race and ethnicity, with the highest proportion of Black and Asian/Other patients undergoing surgical resection (17.0% and 19.4%, respectively; *p* < 0.0001). Ultimately, 14.3% of patients received liver transplants (18.8%, 17.4%, 6.9%, and 4.9% of White, Hispanic, Asian/Other, and Black patients, respectively; *p* < 0.0001). A higher proportion of Black, Asian/Other, and Hispanic patients remained untreated compared to White patients (30.1%, 25.0%, 22.5% vs. 18.6%, respectively; *p* < 0.0001).

**TABLE 2:** Tumor burden, stratified by race and ethnicity

	Non-Hispanic White (n = 851)	Hispanic (n = 737)	Non-Hispanic Black (n = 531)	Asian/other (n = 144)	*p* ^a^
AFP level (ng/mL)	21 (5–235)	25 (6–555)	124 (15–2217)	68 (8–1602)	<0.001
Number lesions, n (%)					
1	508 (59.7)	441 (59.8)	285 (53.7)	84 (54.3)	<0.001
2	152 (17.9)	111 (15.1)	76 (14.3)	24 (16.7)	
3	48 (5.6)	61 (8.3)	34 (6.4)	7 (4.9)	
≥4	40 (4.7)	30 (4.1)	18 (3.4)	1 (0.7)	
Infiltrative/innumerable, n (%)	103 (12.1)	93 (12.6)	118 (22.2)	28 (19.4)	
Infiltrative disease, n (%)	99 (11.6)	108 (14.7)	123 (23.2)	27 (18.8)	<0.001
Vascular invasion, n (%)	131 (15.4)	118 (16.0)	154 (29.0)	25 (17.4)	<0.001
Distant metastases, n (%)	57 (6.7)	72 (9.8)	63 (11.9)	14 (9.7)	0.04
Within Milan, n (%)	438 (51.5)	350 (47.5)	205 (38.6)	60 (41.7)	<0.001
ECOG performance, n (%)					
ECOG 0	522 (61.3)	403 (54.7)	317 (59.7)	83 (57.6)	
ECOG 1	243 (28.6)	228 (30.9)	151 (28.4)	45 (31.3)	0.37
ECOG ≥2	85 (10.0)	104 (14.1)	61 (11.4)	16 (11.1)	
Missing	1 (0.1)	2 (0.3)	2 (0.4)	0 (0.0)	
BCLC tumor stage, n (%)					
BCLC 0/A	389 (45.7)	302 (41.0)	196 (36.9)	63 (43.8)	
BCLC B	195 (22.9)	138 (18.7)	102 (19.2)	32 (22.2)	<0.001
BCLC C	134 (15.8)	137 (18.6)	162 (30.5)	36 (25.0)	
BCLC D	130 (15.3)	160 (21.7)	68 (12.8)	13 (9.0)	
Most definitive treatment, n (%)					<0.001
Transplant	160 (18.8)	128 (17.4)	26 (4.9)	10 (6.9)	
Resection	119 (14.0)	62 (8.4)	90 (17.0)	28 (19.4)	
Ablation	154 (18.1)	117 (15.9)	58 (10.9)	16 (11.1)	
TACE/TARE/SBRT	204 (24.0)	182 (24.7)	116 (21.9)	38 (26.4)	
Systemic therapy	56 (6.6)	82 (11.1)	81 (51.3)	16 (11.1)	
No treatment	158 (18.6)	166 (22.5)	160 (30.1)	36 (25.0)	

a Comparisons were made using ANOVA and chi-square tests for continuous and categorical variables, respectively.

Abbreviations: AFP, alpha-fetoprotein; BCLC, Barcelona Clinic Liver Cancer; ECOG, Eastern Cooperative Oncology Group score; SBRT, stereotactic body radiation therapy; TACE, transarterial chemoembolization; TARE, transarterial radioembolization.

**FIGURE 1:**
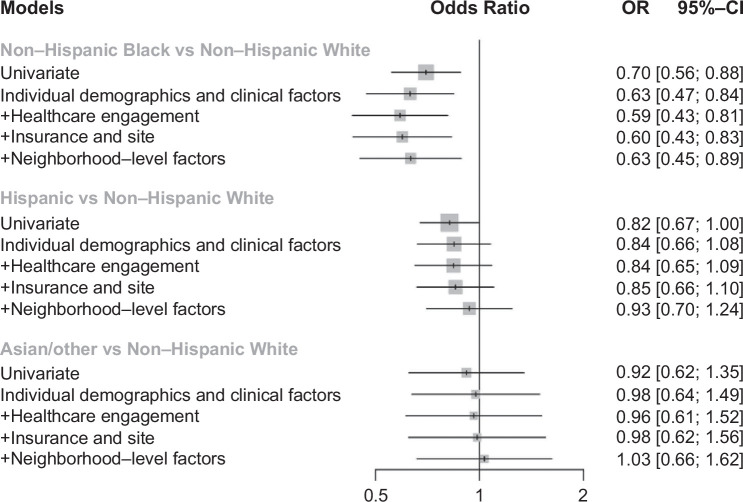
Odds of BCLC early-stage presentation by race and ethnicity (models 1–4). Individual demographic and clinical factors included age, sex, race, ethnicity, smoking, alcohol use, presence of diabetes, CirCom comorbidity score, liver disease etiology, and Child Pugh score. Healthcare engagement factors included PCP visit within 1 year prior to HCC diagnosis and hepatology visit within 1 year prior to HCC diagnosis. Insurance and site included type of insurance (or lack of insurance) as well as hospital site. Neighborhood-level factors included nSES (Yost quintile) and residential segregation measure (LQRRS). Abbreviations: BCLC, Barcelona Clinic Liver Cancer; nSES, neighborhood socioeconomic status; PCP, primary care providern.

In univariable analysis, race and ethnicity were statistically significantly associated with early-stage HCC, with Black (OR: 0.70, 95% CI: 0.56–0.88) and Hispanic patients (OR: 0.82, 95% CI: 0.67–0.99) less likely to be diagnosed at BLC stage 0/A compared to White patients. Results from nested multivariable models for early-stage HCC diagnosis are illustrated in Figure 1. After adjusting for individual demographics, health behaviors, and clinical factors (model 1), Black patients (OR: 0.68, 95% CI: 0.47–0.84) remained significantly less likely than White patients to present with BCLC stage 0/A HCC, whereas early-stage presentation did not significantly differ between Hispanic and White patients (OR: 0.84, 95% CI: 0.66–1.08) or between Asian/Other and White patients (OR: 0.86, 95% CI: 0.59–1.26). Black-White disparities in early-stage detection persisted with the addition of covariates over the 4 models (OR: 0.65, 95% CI: 0.45–0.89 in the fully adjusted model). Conversely, Hispanic-White disparities in early-stage presentation were further mitigated after adjusting for insurance and site (model 3: OR: 0.85, 95% CI: 0.66–1.10) and neighborhood social determinants of health (nSES and LQRRS) in the fully adjusted model (model 4: OR: 0.93, 95% CI: 0.70–1.24). There was no significant difference in early-stage presentation between White and Asian/Other patients in any of the models (model 4: OR: 1.03, 95% CI: 0.66–1.62).

In a sensitivity analysis using the Milan Criteria, 46.7% of the cohort presented with early-stage HCC. Fewer Black patients presented within the Milan Criteria than White, Hispanic, and Asian/Other patients (38.8% vs. 51.7%, 47.5%, and 41.7%, respectively; *p* < 0.001). In univariable analysis, compared to White patients, Black patients (OR: 0.60, 95% CI: 0.48–0.75) and Asian/Other patients (OR: 0.67, 95% CI: 0.47–0.94) had lower odds, whereas Hispanic patients had similar odds (OR: 0.85, 95% CI: 0.66–1.08) of early-stage HCC. Nested multivariable models are shown in Supplemental Figure S1, Supplemental Digital Content 1, http://links.lww.com/HC9/A948. Black-White disparities in early-stage presentation persisted across all models (model 4: OR: 0.63, 95% CI: 0.45–0.86). The disparity between Asian/Other and White patients was no longer observed after adjusting for individual demographics and clinical factors, including liver dysfunction (model 2: OR: 0.78, 95% CI: 0.53–1.14). In fully adjusted models, Hispanic patients (OR: 0.92, 95% CI: 0.69–1.23) and Asian/Other patients (OR: 0.81, 95% CI: 0.54–1.22) had no statistically significant difference in early-stage presentation compared to White patients.

### Overall survival

**FIGURE 2:**
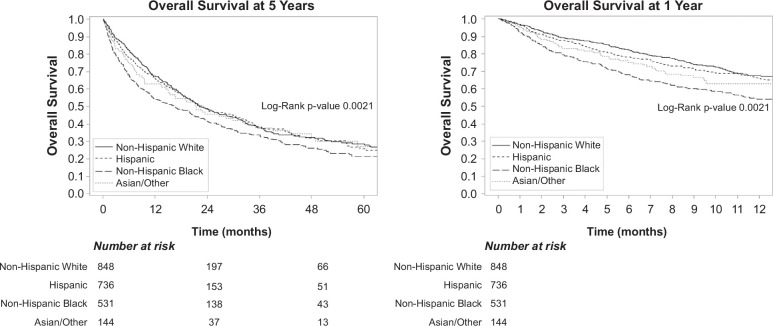
Overall survival at 5 years and 1 year, stratified by race and ethnicity.

A minority of patients underwent curative therapy, and the median overall survival of the cohort was 14.5 (IQR: 4.4–34.2) months, with 1-year and 3-year survival of 65.7% and 44.3%, respectively. Overall survival, stratified by race and ethnicity, is illustrated in Figure 2. For White patients, the median overall survival was 16.2 (IQR: 5.8–36.8) months, compared to 15.7 (IQR: 4.6–34.4) months for Hispanic, 10.0 (IQR: 2.9–29.0) months for Black, and 9.5 (IQR: 3.4–31.9) months for Asian/Other patients (*p* = 0.002).

In univariable analysis, compared to White patients, Black patients had worse survival (HR: 1.30, 95% CI: 1.12–1.51), but Hispanic patients (HR: 1.02, 95% CI: 0.87–1.19) and Asian/Other (HR: 1.07, 95% CI: 0.85–1.33) had similar survival. In stage-stratified analyses (Supplemental Table S1, Supplemental Digital Content 2, http://links.lww.com/HC9/A948), Black-White disparities in survival were observed for those with BCLC stage D tumors (HR: 2.27, 95% CI: 1.57–3.29) but not BCLC stages 0/A (HR: 1.01; 95% CI: 0.76–1.33), B (HR: 0.92, 95% CI: 0.67–1.27), or C (HR: 1.23, 95% CI: 0.95–1.60) HCC. Survival did not significantly differ between Hispanic and White patients across all BCLC stages.

**FIGURE 3:**
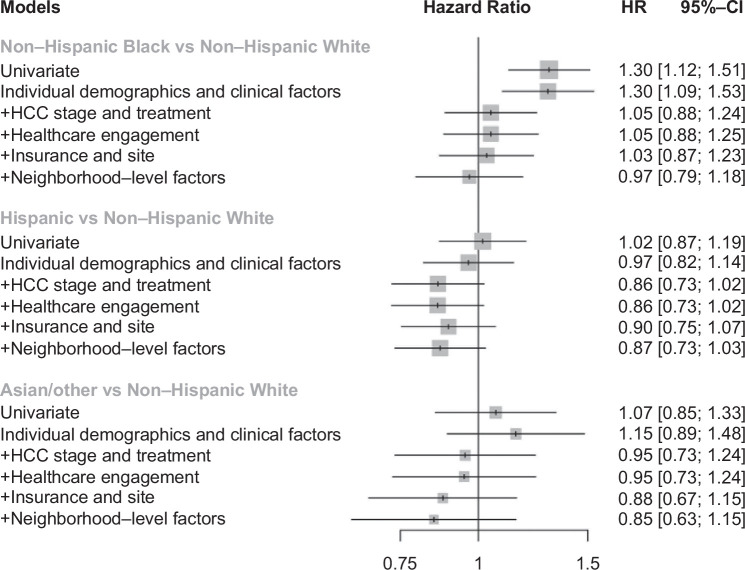
Hazard ratios for death by race and ethnicity (models 1–5). Individual demographic and clinical factors included age, sex, race, ethnicity, smoking, alcohol use, presence of diabetes, CirCom comorbidity score, liver disease etiology, and Child Pugh score. HCC stage and treatment included BCLC stage and HCC treatment modality received. Healthcare engagement factors included PCP visit within 1 year prior to HCC diagnosis and hepatology visit within 1 year prior to HCC diagnosis. Insurance and site included type of insurance (or lack of insurance) as well as hospital site. Neighborhood-level factors included nSES (Yost quintile) and residential segregation measure (LQRRS). Abbreviations: BCLC, Barcelona Clinic Liver Cancer; nSES, neighborhood socioeconomic status; PCP, primary care providern.

Black-White disparities in survival persisted after adjusting for individual demographics and clinical factors (model 2: HR: 1.30, 95% CI: 1.09–1.53); however, they were no longer observed after the introduction of HCC stage and treatment (model 3: HR: 1.05, 95% CI: 0.88–1.24], or in the fully adjusted models (model 5: HR: 0.97, 95% CI: 0.79–1.18) (Figure 3). In fully adjusted models, there continued to be no Hispanic-White disparities (HR: 0.87, 95% CI: 0.73–1.03) or differences in survival between Asian/Other and White patients (HR: 0.85, 95% CI: 0.63–1.15). However, nSES was significantly associated with overall survival (model 5: Yost Quintile 5 vs. Quintile 1: HR: 0.69, 95% CI: 0.48–0.99). Complete nested model results are presented in Supplemental Table S2, Supplemental Digital Content 3, http://links.lww.com/HC9/A948.

## DISCUSSION

In this large, racially and ethnically diverse population of patients diagnosed with HCC across 4 health systems, less than half of patients presented at an early stage, and median survival remained poor at only 16 months. We observed racial and ethnic disparities in HCC early-stage diagnosis and survival, with Black and Hispanic patients being less likely to be diagnosed at an early stage and Black patients having worse overall survival than White patients. However, ethnic disparities in early detection and Black-White disparities in survival were mitigated after accounting for tumor stage, HCC treatment receipt, insurance, and nSES. These data highlight the importance of considering intersectionality when examining health disparities and identifying appropriate intervention targets to improve outcomes.^25^

Consistent with previous studies, we found Black-White and Hispanic-White disparities in early-stage presentation.^18^,^26^ Black-White disparities in early-stage presentation persisted in fully adjusted models, although Hispanic-White and Asian/other-White disparities disappeared after adjusting for individual demographics and clinical factors, including liver function, and were further mitigated when adjusting for health care engagement and neighborhood-level factors. These data suggest that ethnic differences in early-stage disease are likely partly related to health care access to subspecialty care and HCC surveillance. Surveillance is consistently associated with early detection but remains underused in practice—particularly among minoritized and low SES populations.^5^,^27^ The persistence of Black-White disparities in fully adjusted models bears further investigation. There are conflicting data about differences in the sensitivity of ultrasound and alpha-fetoprotein for early-stage HCC^28^,^29^; however, there may be differences in downstream processes, including timely diagnostic evaluation after a positive surveillance result.^30^,^31^ Differences in the completion of these downstream care processes can be influenced by several factors that were not measured in this study, including health literacy, medical mistrust, and health locus of control.^32^ Therefore, future studies prospectively measuring these types of factors are needed to evaluate racial disparities in early HCC presentation.

Our findings regarding disparities in overall survival contrast those reported by others. Many prior studies have consistently demonstrated worse survival in Black patients with HCC compared to other groups^7^,^9^,^18^,^26^; however, most were conducted in population-based cohorts and cancer registries lacking granular clinical information (eg, liver function, liver disease etiology, access to health care, and behavioral factors). These factors are critical to capture as they may act as confounders or mediators of survival disparities. We observed Black-White differences in survival in univariable analysis, although disparities were no longer observed in fully adjusted models accounting for tumor stage, HCC treatment, health care access, and nSES. This pattern suggests Black-White disparities in HCC survival may be driven by differences in insurance, access to care, health system-level factors, or even neighborhood-level factors—which, once again, were not measured in most prior studies. Indeed, insurance status can impact access to HCC surveillance, liver-specific care, including antiviral treatment, and HCC treatment.^33^,^34^ We also observed that Hispanic patients with HCC had similar or better survival compared to non-Hispanic White and non-Hispanic Black patients; prior studies are conflicting on the magnitude and direction of mortality differences in Hispanic patients with HCC compared to their non-Hispanic counterparts.^7^,^35^,^36^ This survival advantage is consistent with the epidemiologic phenomenon previously described in other cancers and chronic diseases as the “Hispanic mortality paradox,” where despite facing socioeconomic disadvantage and having relatively less access to health care in the United States, Hispanic individuals have similar or better health outcomes than other racial and ethnic groups, including non-Hispanic Whites.^37^ However, it remains unclear whether this survival advantage is primarily related to risk and/or resilience factors (eg, diet, lower rates of smoking, strong family, and social support), and further studies are needed to determine the relative impact of biological, psychological, behavioral, and social/cultural factors on the heterogeneity of health outcomes across racial and ethnic groups.

Financial toxicity of cancer care has been well reported in other cancers,^38^ and recent studies have highlighted significant increased patient liabilities related to HCC beyond costs of underlying cirrhosis.^39^ Financial barriers to cancer-related care appear greater in racial and ethnic minority populations than non-Hispanic White patients.^40^ Similarly, prior studies have shown that site-level factors, including patient volume,^41^ type of health care system (eg, safety-net status),^42^ and the presence of multidisciplinary care^43^ are each significantly associated with differences in HCC prognosis. Finally, a SEER-Medicare database analysis found significantly worse survival among Black patients in high-poverty neighborhoods (HR: 1.13, 95% CI: 1.02–1.25) but not in low-poverty neighborhoods (HR: 0.87, 95% CI: 0.73–1.04) compared to White patients,^9^ highlighting the importance of accounting for neighborhood-level factors. SES and insurance coverage are particularly relevant for patients being considered for liver transplantation, which may explain why racial disparities were most marked in those with BCLC stage D tumors. Prior studies have highlighted Black patients are less likely than White patients to be referred for liver transplantation.^44^,^45^

Overall, our data highlight the importance of considering broader factors—at the patient, health system, and neighborhood levels—when developing interventions to improve HCC-related disparities. Structural and large-scale policy changes are needed and they begin by raising public awareness of racial and ethnic disparities in care and identifying actionable interventions to address health equity. These may include insurance reform, improving the capacity and number of providers in underserved communities (both primary care and subspecialists), and addressing poverty and other social determinants of health. Improving access to care (eg, through health policy, universal health care, and expanding health insurance coverage), implementing economic security programs to reduce poverty, and interventions to reduce financial toxicity in cancer care may be the most meaningful steps in reducing HCC disparities in the United States. In addition, neighborhood-level interventions to address food insecurity, lack of green space, and crime may help address some of the nSES disparities observed in this study. Finally, studies from other diseases have highlighted provider-level factors contributing to disparities, such as implicit bias, which remains understudied in HCC.

We acknowledge the limitations of our study. First, we analyzed outcomes among a retrospective cohort of patients with HCC; thus, there are inherent limitations, including missing data, measurement bias, and unmeasured confounders. For example, patients may have had clinic visits or HCC surveillance completed outside the health system, which are not routinely captured. However, we included several covariates not readily available in other studies (eg, liver dysfunction, performance status, comorbidity, socioeconomic status, and neighborhood factors). Second, as with other studies using census tract-level variables for SES rather than individual-level SES data, our results are prone to ecological and individualistic fallacy, that is, incorrect inferences being made about individuals based on characteristics observed among an entire group.^46^ Third, while we classified race, ethnicity, and sex into discrete categories for our analysis, race is a complex social construct, with the direct and indirect impacts of racism varying across time periods and regions. This is of particular importance given the shifting demographics of the United States and the sharp increase in individuals identifying themselves as multiracial.^47^ In addition, there is often greater genetic variation among individuals within the same racial or ethnic group compared to individuals belonging to different groups; thus, these groups cannot be considered as a monolith. Hispanic individuals and Asian individuals are often combined into a single group for data analyses, but it is important to recognize there are many subgroups within these groups with important cultural and socioeconomic differences that can differentially impact health outcomes. For example, the country of origin/ancestry of the Hispanic population in Texas is predominantly from Mexico, whereas a higher proportion of Hispanic individuals in Florida are from Cuba, Puerto Rico, or the Dominican Republic.^48^ Fourth, we acknowledge that race and SES are highly correlated, and we did not examine statistical interaction. Fifth, we had a limited number of Asian patients in this study and thus were likely underpowered to detect differences in this group compared to others. Sixth, despite performing manual chart reviews at both sites, including attempting to access outside records through CareEverywhere or elsewhere in the EHR, less than one-third of patients were documented to have had prior care within the 1 year before HCC diagnosis at our health systems, reflecting a high proportion of patients having fragmented care.^49^ We did not capture whether patients were referred to HCC clinics internally or externally (ie, from the community) but we have previously demonstrated that unfortunately >50% of patients with HCC, particularly those from low SES backgrounds, present new to the health care system with minimal outpatient engagement in clinical care until symptomatic or detected incidentally.^50^ Finally, although our study included tertiary care and safety-net health systems in different geographic regions of the United States, our results may not be generalizable to other types of US health systems and those in other geographic locations, including non-US sites. Overall, we believe these weaknesses are outweighed by the study’s notable strengths, including its large, diverse cohort from different types of health systems, incorporation of granular clinical features missing from prior analyses on this topic, and linkage to neighborhood covariates.

In summary, we found racial, ethnic, and socioeconomic disparities in HCC prognosis, including early HCC presentation and overall survival. Specifically, Black-White disparities for early tumor detection and socioeconomic disparities in overall survival persisted in fully adjusted models. Black-White disparities in survival may be driven by differences in early detection and treatment receipt, and nSES was associated with survival. Despite having lower rates of early tumor detection, Hispanic and Asian/other patients had similar survival compared to White patients. Further prospective studies should continue to tailor and refine potential intervention targets for various populations to reduce sociodemographic disparities in the future.

## Supplementary Material
