# Supplementary material for: Impact of racial, ethnic, and socioeconomic disparities on presentation and survival of HCC: A multicenter study
Source: Hepatol Commun. 2024 Oct 10;8(11):e0477. doi: 10.1097/HC9.0000000000000477 (PMC11469814; doi:10.1097/HC9.0000000000000477)
Supplement: Supplementary file 1 [file hc9-8-e0477-s001.docx]

**Supplemental Figure 1.** Odds of early-stage presentation (defined using Milan criteria) by race and ethnicity (Models 1-4)

**
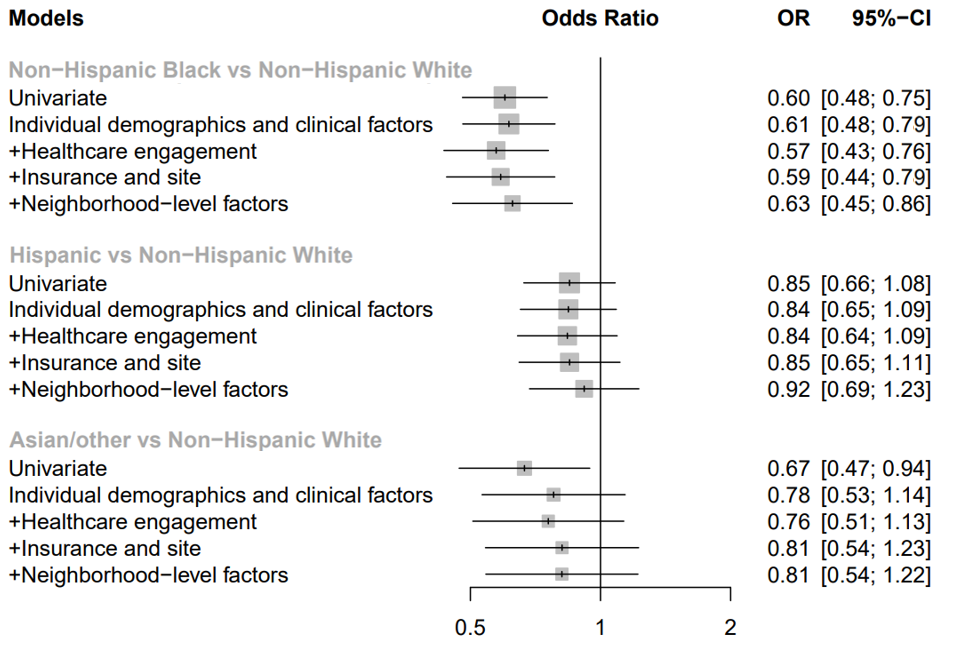
**

Model 1: Age, sex, race and ethnicity, smoking, alcohol use, presence of diabetes, CirCom comorbidity score, liver disease etiology, Child Pugh score

Model 2: Model 1 + PCP visit 1 year prior, Hepatology visit 1 year prior

Model 3: Model 2 + hospital site and insurance

Model 4: Model 3 + nSES (Yost quintile) and residential segregation (LQRRS)

**Supplemental Table 1.** Stage-stratified survival

| **BCLC Stage** | **Race/Ethnicity** | **Hazard Ratio** | **95% CI** |
| --- | --- | --- | --- |
| **A** | **Non-Hispanic White** | Ref |  |
|  | **Non-Hispanic Black** | 1.01 | (0.76, 1.33) |
|  | **Hispanic** | 0.83 | (0.64, 1.09) |
|  | **Asian/other** | 0.85 | (0.55, 1.31) |
| **B** | **Non-Hispanic White** | Ref |  |
|  | **Non-Hispanic Black** | 0.92 | (0.67, 1.27) |
|  | **Hispanic** | 0.93 | (0.69, 1.25) |
|  | **Asian/other** | 1.37 | (0.82, 2.28) |
| **C** | **Non-Hispanic White** | Ref |  |
|  | **Non-Hispanic Black** | 1.23 | (0.95, 1.60) |
|  | **Hispanic** | 1.00 | (0.75, 1.33) |
|  | **Asian/other** | 1.36 | (0.89, 2.07) |
| **D** | **Non-Hispanic White** | Ref |  |
|  | **Non-Hispanic Black** | 2.27 | (1.57, 3.29) |
|  | **Hispanic** | 1.19 | (0.86, 1.66) |
|  | **Asian/other** | 1.28 | (0.55, 2.98) |

**Supplemental Table 2.** Complete nested model results for A) BCLC early stage detection; B) Milan early stage detection and C) Overall survival

1. BCLC early stage detection

**Model 1 (Individual demographics, clinical factors, health behaviors)**

| **Effect** | **Odds Ratio** | **95% Confidence Limits** | |
| --- | --- | --- | --- |
| AgeatHCCdiagnosis | 0.98373 | 0.97425 | 0.99329 |
| Gender Male vs Female | 0.65631 | 0.53591 | 0.80375 |
| Race - Asian/other vs Non-Hispanic White | 0.97603 | 0.63714 | 1.49517 |
| Race - Hispanic White vs Non-Hispanic White | 0.84391 | 0.66092 | 1.07756 |
| Race - Non-Hispanic Black vs Non-Hispanic White | 0.63002 | 0.47046 | 0.8437 |
| Smoking Active vs None | 0.92275 | 0.70431 | 1.20893 |
| Smoking Quit vs None | 0.91387 | 0.7267 | 1.14924 |
| Smoking Unknown/Not Reported vs None | 1.15733 | 0.63077 | 2.12348 |
| Alcohol use Current/Prior heavy use vs None | 0.84116 | 0.65848 | 1.07452 |
| Alcohol use Current social use vs None | 0.66191 | 0.49474 | 0.88556 |
| Diabetes Yes vs No | 1.05286 | 0.86118 | 1.2872 |
| Cirrhosis Comorbidity score >=1 vs 0 | 1.05947 | 0.87482 | 1.2831 |
| Liver disease - Cryptogenic vs HCV Viremic | 0.37536 | 0.19838 | 0.71021 |
| Liver disease - EtOH vs HCV Viremic | 1.3016 | 1.00032 | 1.69362 |
| Liver disease - HBV vs HCV Viremic | 0.49209 | 0.33445 | 0.72402 |
| Liver disease - HCV post-SVR vs HCV Viremic | 1.6873 | 1.26698 | 2.24706 |
| Liver disease - MASLD vs HCV Viremic | 1.17322 | 0.77142 | 1.7843 |

**Model 2 (Model 1+ Healthcare engagement)**

| **Effect** | **Odds Ratio** | **95% Confidence Limits** | |
| --- | --- | --- | --- |
| AgeatHCCdiagnosis | 0.98516 | 0.97564 | 0.99477 |
| Gender Male vs Female | 0.70665 | 0.57223 | 0.87264 |
| Race - Asian/other vs Non-Hispanic White | 0.96358 | 0.61252 | 1.51584 |
| Race - Hispanic White vs Non-Hispanic White | 0.84071 | 0.65048 | 1.08656 |
| Race - Non-Hispanic Black vs Non-Hispanic White | 0.58902 | 0.42844 | 0.80981 |
| Smoking Active vs None | 0.95075 | 0.72757 | 1.2424 |
| Smoking Quit vs None | 0.91371 | 0.72483 | 1.15181 |
| Smoking Unknown/Not Reported vs None | 1.32895 | 0.72423 | 2.43858 |
| Alcoholuse Current/Prior heavy use vs None | 0.75064 | 0.57855 | 0.97391 |
| Alcoholuse Current social use vs None | 0.66735 | 0.4956 | 0.89861 |
| Diabetes Yes vs No | 0.97157 | 0.79209 | 1.19173 |
| Cirrhosis Comorbidity score >=1 vs 0 | 0.87463 | 0.71828 | 1.065 |
| Liver disease - Cryptogenic vs HCV Viremic | 0.38093 | 0.20024 | 0.72468 |
| Liver disease - EtOH vs HCV Viremic | 1.34963 | 1.02752 | 1.77272 |
| Liver disease - HBV vs HCV Viremic | 0.47963 | 0.32307 | 0.71205 |
| Liver disease - HCV post-SVR vs HCV Viremic | 1.74451 | 1.29917 | 2.34251 |
| Liver disease - MASLD vs HCV Viremic | 1.19121 | 0.77441 | 1.83233 |
| Liver disease - Other vs HCV Viremic | 0.38092 | 0.24764 | 0.58592 |
| Child Pugh score | 0.69237 | 0.65527 | 0.73158 |
| pcp 1 vs 0 | 1.40516 | 1.0756 | 1.8357 |
| hep 1 vs 0 | 2.55365 | 1.95683 | 3.33249 |

**Model 3 (Model 2 + site and insurance)**

| **Effect** | **Odds Ratio** | **95% Confidence Limits** | |
| --- | --- | --- | --- |
| Ageat HCCdiagnosis | 0.98166 | 0.97097 | 0.99247 |
| Gender Male vs Female | 0.69524 | 0.56518 | 0.85524 |
| Race - Asian/other vs Non-Hispanic White | 0.98471 | 0.62347 | 1.55527 |
| Race - Hispanic White vs Non-Hispanic White | 0.85185 | 0.65888 | 1.10134 |
| Race - Non-Hispanic Black vs Non-Hispanic White | 0.59829 | 0.4305 | 0.83147 |
| Smoking Active vs None | 0.99041 | 0.76016 | 1.29038 |
| Smoking Quit vs None | 0.91391 | 0.72475 | 1.15245 |
| Smoking Unknown/Not Reported vs None | 1.17204 | 0.61485 | 2.23418 |
| Alcohol use Current/Prior heavy use vs None | 0.7719 | 0.59321 | 1.00442 |
| Alcoholuse Current social use vs None | 0.6651 | 0.49219 | 0.89876 |
| Diabetes Yes vs No | 0.9576 | 0.77674 | 1.18057 |
| Cirrhosis Comorbidity score >=1 vs 0 | 0.93094 | 0.74925 | 1.15669 |
| Liver disease - Cryptogenic vs HCV Viremic | 0.39367 | 0.2047 | 0.75706 |
| Liver disease - EtOH vs HCV Viremic | 1.32458 | 1.004 | 1.74751 |
| Liver disease - HBV vs HCV Viremic | 0.47212 | 0.3149 | 0.70784 |
| Liver disease - HCV post-SVR vs HCV Viremic | 1.66695 | 1.23076 | 2.25774 |
| Liver disease - MASLD vs HCV Viremic | 1.21491 | 0.79362 | 1.85984 |
| Liver disease - Other vs HCV Viremic | 0.37849 | 0.24464 | 0.58558 |
| Child Pugh score | 0.69074 | 0.65366 | 0.72991 |
| pcp 1 vs 0 | 1.50839 | 1.12805 | 2.01697 |
| hep 1 vs 0 | 2.70021 | 2.06735 | 3.52681 |
| Hospital Jackson vs PHHS | 1.22243 | 0.90282 | 1.6552 |
| Hospital UTSW vs PHHS | 0.87535 | 0.60407 | 1.26844 |
| Hospital University of Miami (UM) vs PHHS | 1.03161 | 0.73158 | 1.4547 |
| Insurance Medicaid vs Medicare | 0.75469 | 0.57686 | 0.98735 |
| Insurance Other vs Medicare | 0.72032 | 0.52345 | 0.99123 |
| Insurance Private vs Medicare | 0.9873 | 0.74516 | 1.30813 |
| Insurance Uninsure vs Medicare | 0.73249 | 0.45849 | 1.17024 |
| Insurance Unknown/ vs Medicare | 0.57532 | 0.10818 | 3.05954 |

**Model 4 (Model 3 + neighborhood level factors)**

| **Effect** | **Odds Ratio** | **95% Confidence Limits** | |
| --- | --- | --- | --- |
| AgeatHCCdiagnosis | 0.98526 | 0.97363 | 0.99702 |
| Gender Male vs Female | 0.6455 | 0.52113 | 0.79954 |
| Race - Asian/other vs Non-Hispanic White | 1.03363 | 0.659 | 1.62121 |
| Race - Hispanic White vs Non-Hispanic White | 0.93434 | 0.70252 | 1.24265 |
| Race - Non-Hispanic Black vs Non-Hispanic White | 0.63223 | 0.45106 | 0.88617 |
| Smoking Active vs None | 1.09703 | 0.83042 | 1.44924 |
| Smoking Quit vs None | 0.98079 | 0.76854 | 1.25167 |
| Smoking Unknown/Not Reported vs None | 1.29941 | 0.65726 | 2.56892 |
| Alcoholuse Current/Prior heavy use vs None | 0.8057 | 0.60639 | 1.07052 |
| Alcoholuse Current social use vs None | 0.62972 | 0.46463 | 0.85346 |
| Diabetes Yes vs No | 0.93613 | 0.75413 | 1.16207 |
| Cirrhosis Comorbidity score >=1 vs 0 | 0.88184 | 0.69816 | 1.11385 |
| Liver disease - Cryptogenic vs HCV Viremic | 0.33315 | 0.16753 | 0.6625 |
| Liver disease - EtOH vs HCV Viremic | 1.34009 | 1.00209 | 1.79208 |
| Liver disease - HBV vs HCV Viremic | 0.45856 | 0.29852 | 0.70437 |
| Liver disease - HCV post-SVR vs HCV Viremic | 1.70663 | 1.23877 | 2.35119 |
| Liver disease - MASLD vs HCV Viremic | 1.1535 | 0.73862 | 1.80142 |
| Liver disease - Other vs HCV Viremic | 0.40172 | 0.25819 | 0.62503 |
| Child Pugh score | 0.68153 | 0.64367 | 0.72162 |
| pcp 1 vs 0 | 1.4529 | 1.06109 | 1.98939 |
| hep 1 vs 0 | 2.59977 | 1.94792 | 3.46976 |
| Hospital Jackson vs PHHS | 1.11332 | 0.79694 | 1.55531 |
| Hospital UTSW vs PHHS | 0.78396 | 0.51777 | 1.18698 |
| Hospital University of Miami (UM) vs PHHS | 0.94174 | 0.63272 | 1.40169 |
| Insurance Medicaid vs Medicare | 0.78662 | 0.59544 | 1.03918 |
| Insurance Other vs Medicare | 0.75335 | 0.53885 | 1.05325 |
| Insurance Private vs Medicare | 1.08399 | 0.80239 | 1.46441 |
| Insurance Uninsure vs Medicare | 0.81603 | 0.48476 | 1.37368 |
| Insurance Unknown/ vs Medicare | 0.6032 | 0.10616 | 3.42732 |
| SES_YostQ 2 vs 1 | 0.90033 | 0.70752 | 1.14567 |
| SES_YostQ 3 vs 1 | 1.31484 | 0.92337 | 1.87226 |
| SES_YostQ 4 vs 1 | 1.24534 | 0.88281 | 1.75674 |
| SES_YostQ 5 vs 1 | 1.0825 | 0.67489 | 1.73632 |
| LQRRS_W | 0.98796 | 0.81393 | 1.19922 |
| LQRRS_B | 0.98604 | 0.88738 | 1.09567 |
| LQRRS_H | 0.97707 | 0.81351 | 1.17352 |

1. Milan Early Detection

**Model 1 (Individual demographics, clinical factors, health behaviors)**

| **Effect** | **Odds Ratio** | **95% Confidence Limits** | |
| --- | --- | --- | --- |
| AgeatHCCdiagnosis | 0.98621 | 0.97633 | 0.9962 |
| Gender Male vs Female | 0.70021 | 0.57238 | 0.8566 |
| Race - Asian/other vs Non-Hispanic White | 0.77901 | 0.5321 | 1.1405 |
| Race - Hispanic White vs Non-Hispanic White | 0.84265 | 0.65263 | 1.088 |
| Race - Non-Hispanic Black vs Non-Hispanic White | 0.61392 | 0.47788 | 0.78868 |
| Smoking Active vs None | 0.88052 | 0.70879 | 1.09385 |
| Smoking Quit vs None | 1.01293 | 0.81311 | 1.26185 |
| Smoking Unknown/Not Reported vs None | 1.52799 | 0.87919 | 2.65556 |
| Alcoholuse Current/Prior heavy use vs None | 0.77561 | 0.61244 | 0.98224 |
| Alcoholuse Current social use vs None | 0.58715 | 0.4599 | 0.74962 |
| Diabetes Yes vs No | 0.99684 | 0.83101 | 1.19576 |
| Cirrhosis Comorbidity score >=1 vs 0 | 0.99931 | 0.84839 | 1.17708 |
| Liver disease - Cryptogenic vs HCV Viremic | 0.36506 | 0.22132 | 0.60215 |
| Liver disease - EtOH vs HCV Viremic | 1.10165 | 0.81746 | 1.48464 |
| Liver disease - HBV vs HCV Viremic | 0.49088 | 0.34453 | 0.6994 |
| Liver disease - HCV post-SVR vs HCV Viremic | 1.597 | 1.24132 | 2.05459 |
| Liver disease - MASLD vs HCV Viremic | 0.95002 | 0.63118 | 1.4299 |
| Liver disease - Other vs HCV Viremic | 0.3587 | 0.23237 | 0.55369 |
| Child Pugh score | 0.93601 | 0.89328 | 0.98079 |

**Model 2 (Model 1+ Healthcare engagement)**

| **Effect** | **Odds Ratio** | **95% Confidence Limits** | |
| --- | --- | --- | --- |
| AgeatHCCdiagnosis | 0.98763 | 0.97769 | 0.99768 |
| Gender Male vs Female | 0.75324 | 0.61182 | 0.92736 |
| Race - Asian/other vs Non-Hispanic White | 0.75728 | 0.5065 | 1.13225 |
| Race - Hispanic White vs Non-Hispanic White | 0.83819 | 0.64281 | 1.09294 |
| Race - Non-Hispanic Black vs Non-Hispanic White | 0.57368 | 0.43427 | 0.75784 |
| Smoking Active vs None | 0.90579 | 0.72453 | 1.1324 |
| Smoking Quit vs None | 1.01751 | 0.81404 | 1.27184 |
| Smoking Unknown/Not Reported vs None | 1.72442 | 0.99685 | 2.98303 |
| Alcoholuse Current/Prior heavy use vs None | 0.69682 | 0.54388 | 0.89277 |
| Alcoholuse Current social use vs None | 0.58968 | 0.46051 | 0.75509 |
| Diabetes Yes vs No | 0.92444 | 0.76598 | 1.11567 |
| Cirrhosis Comorbidity score >=1 vs 0 | 0.82932 | 0.69805 | 0.98528 |
| Liver disease - Cryptogenic vs HCV Viremic | 0.36326 | 0.21506 | 0.61358 |
| Liver disease - EtOH vs HCV Viremic | 1.13256 | 0.84104 | 1.52514 |
| Liver disease - HBV vs HCV Viremic | 0.48351 | 0.33882 | 0.68997 |
| Liver disease - HCV post-SVR vs HCV Viremic | 1.6499 | 1.2754 | 2.13437 |
| Liver disease - MASLD vs HCV Viremic | 0.96206 | 0.63299 | 1.46221 |
| Liver disease - Other vs HCV Viremic | 0.3853 | 0.24978 | 0.59437 |
| Child Pugh score | 0.94118 | 0.89676 | 0.98779 |
| pcp 1 vs 0 | 1.4376 | 1.09233 | 1.89201 |
| hep 1 vs 0 | 2.48696 | 1.95407 | 3.16518 |

**Model 3 (Model 2 + site and insurance)**

| **Effect** | **Odds Ratio** | **95% Confidence Limits** | |
| --- | --- | --- | --- |
| AgeatHCCdiagnosis | 0.9813 | 0.9701 | 0.99262 |
| Gender Male vs Female | 0.73763 | 0.59795 | 0.90995 |
| Race - Asian/other vs Non-Hispanic White | 0.81479 | 0.54182 | 1.22528 |
| Race - Hispanic White vs Non-Hispanic White | 0.84809 | 0.64816 | 1.10967 |
| Race - Non-Hispanic Black vs Non-Hispanic White | 0.58763 | 0.43954 | 0.78562 |
| Smoking Active vs None | 0.97941 | 0.78617 | 1.22014 |
| Smoking Quit vs None | 1.02033 | 0.81748 | 1.27352 |
| Smoking Unknown/Not Reported vs None | 1.34109 | 0.76439 | 2.35289 |
| Alcoholuse Current/Prior heavy use vs None | 0.74229 | 0.57946 | 0.95088 |
| Alcoholuse Current social use vs None | 0.58312 | 0.45387 | 0.74917 |
| Diabetes Yes vs No | 0.89151 | 0.73666 | 1.0789 |
| Cirrhosis Comorbidity score >=1 vs 0 | 0.95562 | 0.78604 | 1.16179 |
| Liver disease - Cryptogenic vs HCV Viremic | 0.38976 | 0.23389 | 0.64951 |
| Liver disease - EtOH vs HCV Viremic | 1.1008 | 0.815 | 1.48681 |
| Liver disease - HBV vs HCV Viremic | 0.46217 | 0.32233 | 0.66267 |
| Liver disease - HCV post-SVR vs HCV Viremic | 1.54676 | 1.18528 | 2.01847 |
| Liver disease - MASLD vs HCV Viremic | 1.01028 | 0.66968 | 1.52412 |
| Liver disease - Other vs HCV Viremic | 0.36077 | 0.23296 | 0.55871 |
| Child Pugh score | 0.93614 | 0.89145 | 0.98307 |
| pcp 1 vs 0 | 1.74149 | 1.29292 | 2.34571 |
| hep 1 vs 0 | 2.79195 | 2.15802 | 3.61212 |
| Hospital Jackson vs PHHS | 1.82484 | 1.31625 | 2.52994 |
| Hospital UTSW vs PHHS | 0.94043 | 0.63098 | 1.40166 |
| Hospital University of Miami (UM) vs PHHS | 1.28243 | 0.88507 | 1.85818 |
| Insurance Medicaid vs Medicare | 0.69467 | 0.53245 | 0.90631 |
| Insurance Other vs Medicare | 0.64097 | 0.48104 | 0.85407 |
| Insurance Private vs Medicare | 0.89211 | 0.67602 | 1.17729 |
| Insurance Uninsure vs Medicare | 0.58845 | 0.38597 | 0.89716 |
| Insurance Unknown/ vs Medicare | 0.88537 | 0.1859 | 4.21679 |

**Model 4 (Model 3 + neighborhood level factors)**

| **Effect** | **Odds Ratio** | **95% Confidence Limits** | |
| --- | --- | --- | --- |
| AgeatHCCdiagnosis | 0.98429 | 0.97209 | 0.99663 |
| Gender Male vs Female | 0.6921 | 0.56094 | 0.85392 |
| Race - Asian/other vs Non-Hispanic White | 0.81415 | 0.543 | 1.2207 |
| Race - Hispanic White vs Non-Hispanic White | 0.91677 | 0.68503 | 1.2269 |
| Race - Non-Hispanic Black vs Non-Hispanic White | 0.62582 | 0.45494 | 0.8609 |
| Smoking Active vs None | 1.07488 | 0.85153 | 1.35682 |
| Smoking Quit vs None | 1.09165 | 0.8663 | 1.37562 |
| Smoking Unknown/Not Reported vs None | 1.51011 | 0.82542 | 2.76276 |
| Alcoholuse Current/Prior heavy use vs None | 0.77425 | 0.59424 | 1.0088 |
| Alcoholuse Current social use vs None | 0.51621 | 0.39882 | 0.66816 |
| Diabetes Yes vs No | 0.87542 | 0.72212 | 1.06127 |
| Cirrhosis Comorbidity score >=1 vs 0 | 0.92077 | 0.75383 | 1.12468 |
| Liver disease - Cryptogenic vs HCV Viremic | 0.3559 | 0.20834 | 0.60797 |
| Liver disease - EtOH vs HCV Viremic | 1.09409 | 0.79798 | 1.50008 |
| Liver disease - HBV vs HCV Viremic | 0.44815 | 0.30556 | 0.65729 |
| Liver disease - HCV post-SVR vs HCV Viremic | 1.57081 | 1.1912 | 2.0714 |
| Liver disease - MASLD vs HCV Viremic | 0.94994 | 0.60969 | 1.48008 |
| Liver disease - Other vs HCV Viremic | 0.35851 | 0.22627 | 0.56803 |
| Child Pugh score | 0.92986 | 0.88661 | 0.97521 |
| pcp 1 vs 0 | 1.66632 | 1.21206 | 2.29082 |
| hep 1 vs 0 | 2.7159 | 2.05135 | 3.59572 |
| Hospital Jackson vs PHHS | 1.81812 | 1.26867 | 2.60554 |
| Hospital UTSW vs PHHS | 0.91474 | 0.58328 | 1.43456 |
| Hospital University of Miami (UM) vs PHHS | 1.24127 | 0.81027 | 1.90153 |
| Insurance Medicaid vs Medicare | 0.72164 | 0.54994 | 0.94695 |
| Insurance Other vs Medicare | 0.67067 | 0.49 | 0.91794 |
| Insurance Private vs Medicare | 0.92367 | 0.68916 | 1.23796 |
| Insurance Uninsure vs Medicare | 0.61953 | 0.3895 | 0.98541 |
| Insurance Unknown/ vs Medicare | 0.93854 | 0.19424 | 4.53488 |
| SES_YostQ 2 vs 1 | 0.88649 | 0.69802 | 1.12583 |
| SES_YostQ 3 vs 1 | 1.48682 | 1.11312 | 1.98599 |
| SES_YostQ 4 vs 1 | 1.40352 | 1.03014 | 1.91224 |
| SES_YostQ 5 vs 1 | 1.42257 | 0.95538 | 2.11823 |
| LQRRS_W | 0.93829 | 0.77409 | 1.13731 |
| LQRRS_B | 0.99532 | 0.8987 | 1.10232 |
| LQRRS_H | 1.02714 | 0.86512 | 1.21951 |

1. Overall Survival

**Model 1 (Individual demographics, clinical factors, health behaviors)**

| AgeatHCCdiagnosis | 1.01073 | 1.00352 | 1.01799 |
| --- | --- | --- | --- |
| Gender Male vs Female | 1.19444 | 1.01517 | 1.40538 |
| Race - Asian/other vs Non-Hispanic White | 1.14994 | 0.89224 | 1.48207 |
| Race - Hispanic White vs Non-Hispanic White | 0.9651 | 0.81636 | 1.14092 |
| Race - Non-Hispanic Black vs Non-Hispanic White | 1.29552 | 1.09408 | 1.53405 |
| Smoking Active vs None | 1.40179 | 1.16393 | 1.68826 |
| Smoking Quit vs None | 1.17759 | 1.01584 | 1.36509 |
| Smoking Unknown/Not Reported vs None | 0.59113 | 0.28455 | 1.22801 |
| Alcoholuse Current/Prior heavy use vs None | 1.05079 | 0.89481 | 1.23397 |
| Alcoholuse Current social use vs None | 1.07426 | 0.86773 | 1.32995 |
| Diabetes Yes vs No | 1.0487 | 0.91062 | 1.20771 |
| Cirrhosis Comorbidity score >=1 vs 0 | 1.16153 | 1.02459 | 1.31677 |
| Liver disease - Cryptogenic vs HCV Viremic | 1.45105 | 1.05239 | 2.00073 |
| Liver disease - EtOH vs HCV Viremic | 0.75679 | 0.61397 | 0.93282 |
| Liver disease - HBV vs HCV Viremic | 1.41775 | 1.14233 | 1.75957 |
| Liver disease - HCV post-SVR vs HCV Viremic | 0.70904 | 0.57858 | 0.86892 |
| Liver disease - MASLD vs HCV Viremic | 0.91666 | 0.71856 | 1.16938 |
| Liver disease - Other vs HCV Viremic | 1.02659 | 0.72335 | 1.45694 |
| Child Pugh score | 1.34828 | 1.29541 | 1.40332 |

**Model 2 (Model 1 + BCLC tumor stage and HCC treatment)**

| **Effect** | **Hazard Ratio** | **95% Confidence Limits** | |
| --- | --- | --- | --- |
| AgeatHCCdiagnosis | 1.00283 | 0.99528 | 1.01044 |
| Gender Male vs Female | 1.16894 | 0.97505 | 1.4014 |
| Race - Asian/other vs Non-Hispanic White | 0.95305 | 0.73443 | 1.23674 |
| Race - Hispanic White vs Non-Hispanic White | 0.86187 | 0.72632 | 1.02272 |
| Race - Non-Hispanic Black vs Non-Hispanic White | 1.04846 | 0.88452 | 1.24278 |
| Smoking Active vs None | 1.2024 | 0.94477 | 1.53029 |
| Smoking Quit vs None | 1.14851 | 0.94242 | 1.39967 |
| Smoking Unknown/Not Reported vs None | 0.95313 | 0.47755 | 1.90231 |
| Alcoholuse Current/Prior heavy use vs None | 0.8565 | 0.71162 | 1.03088 |
| Alcoholuse Current social use vs None | 0.8628 | 0.6982 | 1.06621 |
| Diabetes Yes vs No | 1.01904 | 0.89698 | 1.15771 |
| Cirrhosis Comorbidity score >=1 vs 0 | 1.21269 | 1.03468 | 1.42132 |
| Liver disease - Cryptogenic vs HCV Viremic | 1.47376 | 1.12861 | 1.92447 |
| Liver disease - EtOH vs HCV Viremic | 0.82955 | 0.65816 | 1.04556 |
| Liver disease - HBV vs HCV Viremic | 1.26594 | 0.98445 | 1.62791 |
| Liver disease - HCV post-SVR vs HCV Viremic | 0.89955 | 0.71991 | 1.12402 |
| Liver disease - MASLD vs HCV Viremic | 1.14344 | 0.8512 | 1.536 |
| Liver disease - Other vs HCV Viremic | 0.97827 | 0.70273 | 1.36185 |
| Child Pugh score | 1.14598 | 1.08396 | 1.21154 |
| BCLC Class B vs A | 1.80797 | 1.49384 | 2.18817 |
| BCLC Class C vs A | 3.57376 | 2.87457 | 4.44301 |
| BCLC Class D vs A | 2.3249 | 1.70249 | 3.17486 |
| Most Definitive Treatment Tier - OLT vs No Treatment | 0 | 0 | 0 |
| Most Definitive Treatment Tier - Resection vs No Treatment | 0.1647 | 0.11875 | 0.22844 |
| Most Definitive Treatment Tier - Ablation vs No Treatment | 0.3101 | 0.24625 | 0.3905 |
| Most Definitive Treatment Tier - TACE/TARE/SBRT vs No Treatment | 0.43766 | 0.35301 | 0.54259 |
| Most Definitive Treatment Tier - Chemotherapy vs No Treatment | 0.58166 | 0.44295 | 0.76381 |

**Model 3 (Model 2 + healthcare engagement)**

| **Effect** | **Hazard Ratio** | **95% Confidence Limits** | |
| --- | --- | --- | --- |
| AgeatHCCdiagnosis | 1.00285 | 0.99527 | 1.01049 |
| Gender Male vs Female | 1.17534 | 0.98135 | 1.40769 |
| Race - Asian/other vs Non-Hispanic White | 0.94959 | 0.72813 | 1.23841 |
| Race - Hispanic White vs Non-Hispanic White | 0.86072 | 0.72543 | 1.02125 |
| Race - Non-Hispanic Black vs Non-Hispanic White | 1.04846 | 0.87992 | 1.24929 |
| Smoking Active vs None | 1.20351 | 0.94329 | 1.53551 |
| Smoking Quit vs None | 1.14944 | 0.94263 | 1.40162 |
| Smoking Unknown/Not Reported vs None | 0.95718 | 0.47566 | 1.92615 |
| Alcoholuse Current/Prior heavy use vs None | 0.8529 | 0.70462 | 1.03239 |
| Alcoholuse Current social use vs None | 0.86129 | 0.69563 | 1.06641 |
| Diabetes Yes vs No | 1.0144 | 0.89384 | 1.15122 |
| Cirrhosis Comorbidity score >=1 vs 0 | 1.20371 | 1.01731 | 1.42426 |
| Liver disease - Cryptogenic vs HCV Viremic | 1.47971 | 1.13201 | 1.93419 |
| Liver disease - EtOH vs HCV Viremic | 0.83357 | 0.65814 | 1.05577 |
| Liver disease - HBV vs HCV Viremic | 1.26405 | 0.98171 | 1.6276 |
| Liver disease - HCV post-SVR vs HCV Viremic | 0.90085 | 0.72233 | 1.12349 |
| Liver disease - MASLD vs HCV Viremic | 1.14661 | 0.85587 | 1.53612 |
| Liver disease - Other vs HCV Viremic | 0.98247 | 0.70575 | 1.3677 |
| Child Pugh score | 1.14571 | 1.08374 | 1.21123 |
| BCLC Class B vs A | 1.81676 | 1.5013 | 2.19852 |
| BCLC Class C vs A | 3.59163 | 2.89494 | 4.456 |
| BCLC Class D vs A | 2.33287 | 1.71461 | 3.17408 |
| Most Definitive Treatment Tier - OLT vs No Treatment | 0 | 0 | 0 |
| Most Definitive Treatment Tier - Resection vs No Treatment | 0.16448 | 0.11861 | 0.22809 |
| Most Definitive Treatment Tier - Ablation vs No Treatment | 0.30988 | 0.24609 | 0.39021 |
| Most Definitive Treatment Tier - TACE/TARE/SBRT vs No Treatment | 0.43705 | 0.35299 | 0.54112 |
| Most Definitive Treatment Tier - Chemotherapy vs No Treatment | 0.58148 | 0.44171 | 0.76547 |
| pcp 1 vs 0 | 1.01208 | 0.84554 | 1.21143 |
| hep 1 vs 0 | 1.03536 | 0.85512 | 1.25361 |

**Model 4 (Model 3 + site and insurance)**

| **Effect** | **Hazard Ratio** | **95% Confidence Limits** | |
| --- | --- | --- | --- |
| AgeatHCCdiagnosis | 1.00425 | 0.99543 | 1.01314 |
| Gender Male vs Female | 1.22161 | 1.023 | 1.45878 |
| Race - Asian/other vs Non-Hispanic White | 0.87749 | 0.66728 | 1.15392 |
| Race - Hispanic White vs Non-Hispanic White | 0.89582 | 0.75268 | 1.06617 |
| Race - Non-Hispanic Black vs Non-Hispanic White | 1.03172 | 0.86644 | 1.22853 |
| Smoking Active vs None | 1.19929 | 0.93236 | 1.54265 |
| Smoking Quit vs None | 1.17224 | 0.95994 | 1.43149 |
| Smoking Unknown/Not Reported vs None | 1.19718 | 0.58067 | 2.46824 |
| Alcoholuse Current/Prior heavy use vs None | 0.76069 | 0.62682 | 0.92314 |
| Alcoholuse Current social use vs None | 0.83154 | 0.6714 | 1.02988 |
| Diabetes Yes vs No | 1.043 | 0.91497 | 1.18894 |
| Cirrhosis Comorbidity score >=1 vs 0 | 1.00805 | 0.83926 | 1.21078 |
| Liver disease - Cryptogenic vs HCV Viremic | 1.33772 | 1.01675 | 1.76 |
| Liver disease - EtOH vs HCV Viremic | 0.7921 | 0.61288 | 1.02372 |
| Liver disease - HBV vs HCV Viremic | 1.39569 | 1.08209 | 1.80018 |
| Liver disease - HCV post-SVR vs HCV Viremic | 0.9301 | 0.72442 | 1.19417 |
| Liver disease - MASLD vs HCV Viremic | 1.09277 | 0.82799 | 1.44222 |
| Liver disease - Other vs HCV Viremic | 1.07231 | 0.78769 | 1.45977 |
| Child Pugh score | 1.16819 | 1.10384 | 1.2363 |
| BCLC Class B vs A | 1.92739 | 1.58409 | 2.34509 |
| BCLC Class C vs A | 3.81012 | 3.12601 | 4.64394 |
| BCLC Class D vs A | 2.28518 | 1.64849 | 3.16778 |
| Most Definitive Treatment Tier - OLT vs No Treatment | 0 | 0 | 0 |
| Most Definitive Treatment Tier - Resection vs No Treatment | 0.18417 | 0.13249 | 0.25602 |
| Most Definitive Treatment Tier - Ablation vs No Treatment | 0.35 | 0.27505 | 0.44538 |
| Most Definitive Treatment Tier - TACE/TARE/SBRT vs No Treatment | 0.47094 | 0.37622 | 0.58951 |
| Most Definitive Treatment Tier - Chemotherapy vs No Treatment | 0.60765 | 0.4661 | 0.79219 |
| pcp 1 vs 0 | 0.83909 | 0.68793 | 1.02348 |
| hep 1 vs 0 | 0.93279 | 0.77548 | 1.12201 |
| Hospital Jackson vs PHHS | 0.39882 | 0.27978 | 0.56849 |
| Hospital UTSW vs PHHS | 0.86345 | 0.66516 | 1.12086 |
| Hospital University of Miami (UM) vs PHHS | 0.5727 | 0.4437 | 0.73921 |
| Insurance Medicaid vs Medicare | 0.88085 | 0.69465 | 1.11696 |
| Insurance Other vs Medicare | 0.92049 | 0.72065 | 1.17574 |
| Insurance Private vs Medicare | 0.94042 | 0.78106 | 1.13229 |
| Insurance Uninsure vs Medicare | 1.16488 | 0.87067 | 1.55851 |
| Insurance Unknown/ vs Medicare | 0.4548 | 0.07114 | 2.90749 |

**Model 5 (Model 4 + neighborhood level factors)**

| **Effect** | **Hazard Ratio** | **95% Confidence Limits** | |
| --- | --- | --- | --- |
| AgeatHCCdiagnosis | 1.00492 | 0.996 | 1.01391 |
| Gender Male vs Female | 1.26895 | 1.06808 | 1.5076 |
| Race - Asian/other vs Non-Hispanic White | 0.84902 | 0.62627 | 1.15098 |
| Race - Hispanic White vs Non-Hispanic White | 0.86874 | 0.73433 | 1.02776 |
| Race - Non-Hispanic Black vs Non-Hispanic White | 0.96778 | 0.79371 | 1.18002 |
| Smoking Active vs None | 1.17045 | 0.91859 | 1.49136 |
| Smoking Quit vs None | 1.13172 | 0.92617 | 1.38289 |
| Smoking Unknown/Not Reported vs None | 1.00321 | 0.46823 | 2.14944 |
| Alcoholuse Current/Prior heavy use vs None | 0.72174 | 0.59162 | 0.88048 |
| Alcoholuse Current social use vs None | 0.80759 | 0.64473 | 1.0116 |
| Diabetes Yes vs No | 1.04524 | 0.91597 | 1.19275 |
| Cirrhosis Comorbidity score >=1 vs 0 | 1.0279 | 0.85929 | 1.22959 |
| Liver disease - Cryptogenic vs HCV Viremic | 1.29528 | 0.98319 | 1.70643 |
| Liver disease - EtOH vs HCV Viremic | 0.79994 | 0.61433 | 1.04162 |
| Liver disease - HBV vs HCV Viremic | 1.42667 | 1.09657 | 1.85613 |
| Liver disease - HCV post-SVR vs HCV Viremic | 0.91889 | 0.71186 | 1.18614 |
| Liver disease - MASLD vs HCV Viremic | 1.14022 | 0.86059 | 1.51071 |
| Liver disease - Other vs HCV Viremic | 1.07914 | 0.78032 | 1.4924 |
| Child Pugh score | 1.17231 | 1.10833 | 1.23999 |
| BCLC Class B vs A | 1.92975 | 1.5641 | 2.3809 |
| BCLC Class C vs A | 3.98854 | 3.22454 | 4.93357 |
| BCLC Class D vs A | 2.21795 | 1.594 | 3.08613 |
| Most Definitive Treatment Tier - OLT vs No Treatment | 0 | 0 | 0 |
| Most Definitive Treatment Tier - Resection vs No Treatment | 0.1781 | 0.12683 | 0.25011 |
| Most Definitive Treatment Tier - Ablation vs No Treatment | 0.33887 | 0.26468 | 0.43385 |
| Most Definitive Treatment Tier - TACE/TARE/SBRT vs No Treatment | 0.44721 | 0.35781 | 0.55894 |
| Most Definitive Treatment Tier - Chemotherapy vs No Treatment | 0.57025 | 0.43187 | 0.75297 |
| pcp 1 vs 0 | 0.8544 | 0.70098 | 1.04141 |
| hep 1 vs 0 | 0.96898 | 0.80355 | 1.16848 |
| Hospital Jackson vs PHHS | 0.40816 | 0.28149 | 0.59184 |
| Hospital UTSW vs PHHS | 0.85784 | 0.63877 | 1.15203 |
| Hospital University of Miami (UM) vs PHHS | 0.63307 | 0.47932 | 0.83615 |
| Insurance Medicaid vs Medicare | 0.87816 | 0.68843 | 1.12019 |
| Insurance Other vs Medicare | 0.98199 | 0.7662 | 1.25855 |
| Insurance Private vs Medicare | 0.95004 | 0.77399 | 1.16612 |
| Insurance Uninsure vs Medicare | 1.25254 | 0.91603 | 1.71269 |
| Insurance Unknown/ vs Medicare | 0.45567 | 0.07686 | 2.70138 |
| SES_YostQ 2 vs 1 | 0.95817 | 0.7529 | 1.21939 |
| SES_YostQ 3 vs 1 | 0.95055 | 0.75621 | 1.19484 |
| SES_YostQ 4 vs 1 | 0.83557 | 0.6329 | 1.10315 |
| SES_YostQ 5 vs 1 | 0.68928 | 0.47673 | 0.99662 |
| LQRRS_W | 1.01534 | 0.82282 | 1.2529 |
| LQRRS_B | 1.01227 | 0.92683 | 1.10557 |
| LQRRS_H | 0.90511 | 0.77782 | 1.05322 |
